# A Retrospective Analysis of *Salmonella* Isolates across 11 Animal Species (1982–1999) Led to the First Identification of Chromosomally Encoded *bla*_SCO-1_ in the USA

**DOI:** 10.3390/microorganisms12030528

**Published:** 2024-03-06

**Authors:** Nneka Vivian Iduu, Donna Raiford, Austin Conley, Joy Scaria, Julie Nelson, Laura Ruesch, Stuart Price, Min Yue, Jiansen Gong, Lanjing Wei, Chengming Wang

**Affiliations:** 1 Department of Pathobiology, College of Veterinary Medicine, Auburn University, Auburn, AL 36849, USA; nvi0001@auburn.edu (N.V.I.); drr0004@auburn.edu (D.R.); pricesb@auburn.edu (S.P.); 2 Department of Veterinary Pathobiology, Stillwater, Oklahoma State University, Stillwater, OK 74078, USA; joy.scaria@okstate.edu; 3 Department of Veterinary & Biomedical Sciences, South Dakota State University, Brookings, SD 57007, USA; julie.nelson@sdstate.edu (J.N.); laura.ruesch@sdstate.edu (L.R.); 4 Department of Veterinary Medicine, Zhejiang University, Hangzhou 310027, China; 5 Poultry Institute, Chinese Academy of Agricultural Sciences, Yangzhou 225125, China; jjsensen@163.com; 6 Bioengineering Program, The University of Kansas, Lawrence, KS 66045, USA; lanjingwei@ku.edu

**Keywords:** *Salmonella*, antimicrobial resistance, genomic surveillance, *bla*_SCO-1_

## Abstract

Antimicrobial resistance (AMR) in non-typhoidal *Salmonella* is a pressing public health concern in the United States, necessitating continuous surveillance. We conducted a retrospective analysis of 251 *Salmonella* isolates from 11 animal species recovered between 1982 and 1999, utilizing serotyping, antimicrobial susceptibility testing, and whole-genome sequencing (WGS). Phenotypic resistance was observed in 101 isolates, with *S*. Typhimurium, *S*. Dublin, *S*. Agona, and *S*. Muenster prevailing among 36 identified serovars. Notably, resistance to 12 of 17 antibiotics was detected, with ampicillin being most prevalent (79/251). We identified 38 resistance genes, primarily mediating aminoglycoside (n = 13) and β-lactamase (n = 6) resistance. Plasmid analysis unveiled nine distinct plasmids associated with AMR genes in these isolates. Chromosomally encoded *bla*_SCO-1_ was present in three *S*. Typhimurium and two *S*. Muenster isolates from equine samples, conferring resistance to amoxicillin/clavulanic acid. Phylogenetic analysis revealed three distinct clusters for these five isolates, indicating evolutionary divergence. This study represents the first report of *bla*_SCO-1_ in the USA, and our recovered isolates harboring this gene as early as 1989 precede those of all other reports. The enigmatic nature of *bla*_SCO-1_ prompts further research into its function. Our findings highlight the urgency of addressing antimicrobial resistance in *Salmonella* for effective public health interventions.

## 1. Introduction

*Salmonella*, a Gram-negative Enterobacteriaceae family member, stands as a predominant etiological agent of gastroenteritis [1]. Non-typhoidal *Salmonella* (NTS)*,* an extensive diversity within the *Salmonella* genus, which is evidenced by the documentation of more than 2600 serovars, is the most common enteric pathogen in animals and humans [2]. This poses a substantial and persistent threat to public health and is estimated to cause about 150 million illnesses and 60,000 deaths globally yearly [3]. These different NTS serovars are prevalent across diverse animal hosts, emphasizing the critical intersection between animal and human health [1].

The development of antimicrobial resistance (AMR) within *Salmonella* is attributed to various factors, including chromosomally encoded genes, chromosomal mutations, and the acquisition and recombination of mobile genetic elements such as transposons and plasmids [4,5,6,7]. For instance, *Salmonella* harbors naturally occurring chromosomally mediated β-lactamases, believed to have evolved from penicillin-binding proteins with shared sequence homology, and they have been disseminated by mobile genetic elements [8,9].

The increasing global incidence of AMR in *Salmonella* strains emphasizes the necessity for comprehensive investigations into their genomic complexities to facilitate effective public health interventions [10]. Whole genome sequencing (WGS) has become more accessible in recent years, enabling molecular characterization studies [11]. Determination of the antimicrobial resistance gene (AMG) through WGS complements traditional laboratory-based surveillance, offering insight into *Salmonella* serovars in a high-resolution manner. This approach provides direct insights into their evolutionary changes, strain relatedness, gene location, and detailed gene arrangements [5,12]. Consequently, several studies have established a strong correlation between antimicrobial genotypes and phenotypes in non-typhoidal *Salmonella* [13,14].

Most *Salmonella* genomic surveillance focuses on food animals as carriers, with very few studies addressing diseased animals, creating a general gap in research. One study examined samples from diseased animals due to reported concerns about antimicrobial resistance (AMR) in animals from clinical settings [15,16].

The history of *Salmonella* epidemiology has relied on various features to categorize strains. In vitro antibiotic susceptibility testing remains crucial for monitoring antibiotic resistance trends and guiding effective anti-infective therapy [17]. Thus, this study focused on 251 *Salmonella* clinical isolates collected over 18 years (1982–1999) in the United States from 11 different animal host species. We employed a multidimensional approach encompassing phenotypic characterization and genotypic profiling to understand the complex interplay between genomics and antimicrobial resistance.

The insights garnered from this study are anticipated to inform public health policies and interventions, addressing the diverse evolutionary patterns of antimicrobial resistance in *Salmonella* strains.

## 2. Materials and Methods

### 2.1. Bacterial Isolates

A total of 251 non-typhoidal *Salmonella enterica* clinical isolates of animal origin, recovered over 18 years (1982–1999), were included in this study. All isolates were sourced from the Bacteriology and Mycology Diagnostic Laboratory, College of Veterinary Medicine, at Auburn University. Pure isolates were reactivated by streaking on Tryptic Soy Agar and incubating at 37 °C.

### 2.2. Antimicrobial Susceptibility Testing

The isolates underwent phenotypic characterization via microbroth dilution antimicrobial susceptibility testing using the Vitek^®^ 2 system. The tested antimicrobials (μg/mL) included ampicillin, amoxicillin/clavulanic acid, cefalexin, cefpodoxime, cefovecin, ceftazidime, ceftiofur, imipenem, amikacin, gentamicin, ciprofloxacin, enrofloxacin, marbofloxacin, doxycycline, nitrofurantoin, chloramphenicol, and trimethoprim/sulfamethoxazole. Minimum inhibitory concentration (MIC) values were interpreted using standard guideline breakpoints from the Clinical and Laboratory Standards Institute [18,19], National Antimicrobial Resistance Monitoring System for Enteric Bacteria [20], and IDEXX [21].

### 2.3. Whole-Genome Sequencing (WGS) and Genome Analysis

Genomic DNA extraction was performed on isolates obtained from 1.0 mL of 16–24 h culture prepared in Tryptic Soy Broth using the DNeasy Blood & Tissue Kit (Qiagen, Germantown, MD, USA) according to the manufacturer’s protocol. The quality of isolated DNA was analyzed using NanoDrop™One and quantified using a Qubit^®^ 3.0 fluorometer (Thermo Fisher Scientific, Waltham, MA, USA). The extracted DNA was shipped from Auburn University College of Veterinary Medicine to South Dakota University to perform WGS. A concentration of 0.3 ng/μL of DNA was used for library preparation using Illumina DNA Sample Prep Kit (Illumina Inc., San Diego, CA, USA). After bead normalization, the pooled library was denatured, and sequencing was performed on the Illumina Miseq platform using V3 reagents with 2 × 300 paired-end chemistry. Sequencing data’s basic quality statistics were analyzed by fast QC, and assembly quality details were analyzed by QUAST using the Galaxytrakr platform. The raw sequences were uploaded into NCBI Sequence Read Archive (SRA), and the de novo assembly was generated by running SKESA under the NCBI BioProject PRJNA280335. (https://www.ncbi.nlm.nih.gov/bioproject/?term=PRJNA280335).

Antimicrobial resistance genotypes were predicted using the NCBI Pathogen Detection website (https://www.ncbi.nlm.nih.gov/pathogens/isolates; [22]) and ResFinder (https://cge.food.dtu.dk/services/ResFinder/, [23]). Individual genetic element locations were accessed through Pathogen Detection Microbial Browser for Identification of Genetic and Genomic Elements (MicroBIGG-E) (https://www.ncbi.nlm.nih.gov/pathogens/microbigge/). Plasmids were identified using the PlasmidFinder database on ABRicate (https://github.com/tseemann/abricate, [24,25]) and the plasmid-mediated AMR genes were regarded as genes found sharing the same contigs with identified plasmids [26]. The BlastP analysis was used to identify protein similarities (http://www.ncbi.nlm.nih.gov/BLAST, [27]) and conserved domains of proteins were generated on the NCBI conserved domain platform. (https://www.ncbi.nlm.nih.gov/Structure/cdd/wrpsb.cgi, [28]).

### 2.4. Genotypic and Phenotypic Correlation Analysis

Agreement between phenotypic MIC data and genotypic WGS data was statistically evaluated using Cohen’s kappa (κ) test (https://idostatistics.com/cohen-kappa-free-calculator/#risultati, [29]). Isolates with intermediate resistance were not considered in this analysis. The results were interpreted as 0.01–0.20 slight agreement, 0.21–0.40 fair agreement, 0.41–0.60 moderate agreement, 0.61–0.80 substantial agreement, 0.81–1.00 almost perfect or perfect agreement [30].

### 2.5. Genome Visualization and Analysis

Contigs for the genomes of interest were concatenated, and circular genomes were generated using BLAST Ring Image Generator (BRIG) software (https://sourceforge.net/projects/brig/). Phylogenetic analysis based on Average Nucleotide Identity (ANI) was calculated using MASH clustering to determine percentage similarities among isolates, and the FastANI tool (https://github.com/ParBLiSS/FastANI) was used for the analysis.

## 3. Results

### 3.1. Identification of 36 Salmonella Serovars in 11 Animal Species

Amongst the 251 analyzed isolates of *Salmonella* recovered from 11 animal host species, we identified 36 distinct serovars. Notably, *S*. Typhimurium appeared as the most prevalent, constituting 36.2% (n = 91) of the isolates, followed by *S*. Anatum (9.1%, n = 23), *S*. Dublin (8.3%, n = 21), and *S*. Agona (7.1%, n = 18). Other identified serovars were *S*. Newport (4.3%, n = 11), *S*. Give (3.2%, n = 8), *S*. Mbandaka (3.1%, n = 8), *S*. Muenster (3.1%, n = 8), *S*. Muenchen (2.7%, n = 7), *S*. Enteritidis (2.3%, n = 6), and *S*. Meleagridis (2.0%, n = 5). Additionally, several serovars, such as *S*. Montevideo, *S*. Tennessee, *S*. Worthington, *S*. Cholerasuis, *S*. Uganda, *S*. Berta, and others, exhibited lower prevalence rates (below 2%) (Table S1).

### 3.2. Salmonella Antimicrobial Resistance (AMR) Phenotypes

**Figure 1:**
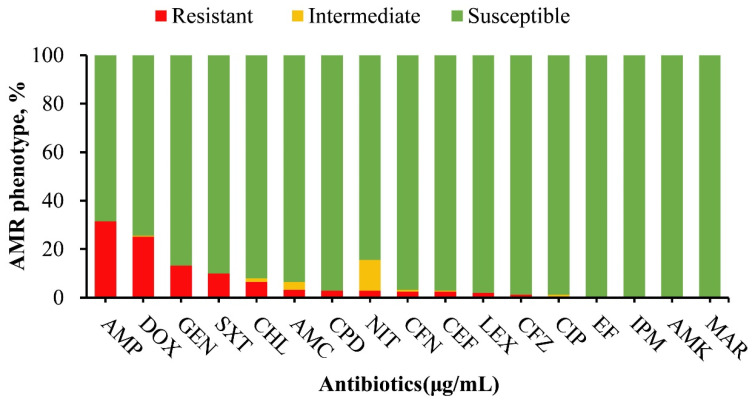
Antimicrobial susceptibility profile of *Salmonella* isolates (n = 251) in this study. The drugs that are mostly resistant are ampicillin (31.5%), doxycycline (25.5%), gentamicin (13.1%), trimethoprim/sulfamethoxazole (10%), and chloramphenicol (6.4%). Other antimicrobials with lower resistance frequencies are AMC (3.2%), CPD (2.8%), NIT (2.8%), CFN (2.8%), CEF (2.8%), LEX (2.0%), CFZ (1.2%). No resistance was observed against CIP, EF, IPM, AMK, MAR. Abbreviations; AMP = ampicillin, AMC = amoxicillin/clavulanic acid, LEX = cephalexin, CPD = cefpodoxime, CFN = cefovecin, CFZ = ceftazidime, CEF = ceftiofur, IPM = imipenem, AMK = amikacin, GEN = gentamicin, CIP = ciprofloxacin, EF = enrofloxacin, MAR = marbofloxacin, DOX = doxycycline, NIT = nitrofurantoin, CHL = chloramphenicol, SXT = trimethoprim/sulfamethoxazole.

**Figure 2:**
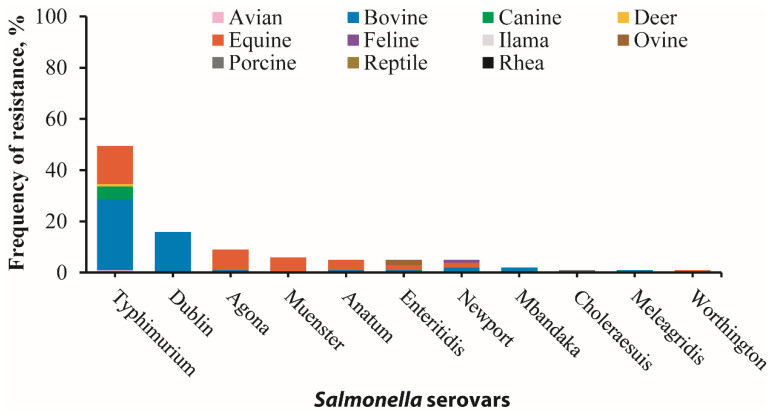
Antimicrobial resistance in 101 *Salmonella* isolates across 11 serovars and 11 animal species exhibiting resistance to at least one antimicrobial tested. The highest frequency of resistance was observed in *S*. Typhimurium (49.5%), followed by *S*. Dublin (15.8%), *S*. Agona (8.9%), *S*. Muenster (5.9%), *S*. Anatum (5.0%), *S*. Enteriditis (5.0%), *S*. Newport (5.0%), *S*. Mbandaka (2.0%), *S*. Choleraesuis (1.0%), *S*. Maleagridis (1.0%), and *S*. Worthington (1.0%). Additionally, bovine-recovered serovars exhibited the highest frequency of resistance (51.5% total resistance) with *S*. Typhimurium 27.7%, *S*. Dublin 15.8%, *S*. Newport 2.0%, *S*. Mbandaka 2.0%, *S*. Anatum 1.0%, and *S*. Enteritidis 1.0%, followed by equine-recovered serovars (37.6% total resistance), which include *S*. Typhimurium 14.9%, *S*. Agona 7.9%, *S*. Muenster 5.9%, *S*. Anatum 4.0%, *S*. Enteritidis 2.0%, *S*. Newport 2.0%, and *S*. Worthington 1.0%. Other animal-recovered serovars with lower resistance are canine-recovered serovars (5% total resistance in *S*. Typhimurium), ovine-recovered serovars (2% total resistance in *S*. Enteritidis), avian-recovered serovars (1% total resistance *S*. Typhimurium), deer-recovered serovars (1% total resistance *S*. Typhimurium), feline-recovered serovars (1% *S*. Newport), and porcine-recovered serovars (1% total resistance *S*. Choleraesuis). Note: animal species of resistant serovars are distinguished by distinct color shades.

In this study, phenotypic resistance was most notable against ampicillin (79/251), doxycycline (64/251), gentamicin (33/251), trimethoprim/sulfamethoxazole (25/251), and chloramphenicol (16/251), among other antimicrobials (Figure 1). One hundred and one isolates (n = 101) displayed resistance to at least one of the 17 tested antimicrobials, of which none of the isolates exhibited resistance to imipenem, amikacin, ciprofloxacin, enrofloxacin, or marbofloxacin. Among the 101 resistant isolates, *S*. Typhimurium serovars were the most prevalent, constituting 49.5% (50/101), followed by *S*. Dublin 15.8% (16/101), *S*. Agona 8.9% (9/101), and *S*. Muenster 5.9% (6/101). Lower resistance prevalence was observed in *S*. Anatum, *S*. Enteritidis, *S*. Newport, *S*. Mbandaka, *S*. Choleraesuis, *S*. Meleagridis, and *S*. Worthington, while other identified isolates did not show antimicrobial resistance (Figure 2).

Furthermore, bovine-recovered serovars displayed the highest overall frequency of antimicrobial resistance at 51.5%, primarily driven by resistance in *S*. Typhimurium (27.7%) and *S*. Dublin (15.8%). Equine isolates showed a resistance rate of 37.6%, where *S*. Typhimurium (14.9%), *S*. Agona (7.9%), and *S*. Muenster (5.9%) were the predominant contributors (Figure 2; Tables S2 and S3). Conversely, serovars from other animal host species showed lower resistance rates (≤5%) (Figure 2).

### 3.3. Antimicrobial Resistance Genes in Salmonella Isolates

Genome analysis identified thirty-eight different antimicrobial resistance genes distributed across our isolates, and aminoglycosides exhibited the largest gene diversity with 13 distinct resistance genes. We also found six β-lactamase-producing genes, including the rarely reported gene *bla*_SCO-1_. AMGs were also identified to mediate resistance to tetracyclines (n = 4), phenicol (n = 3), trimethoprim (n = 3), sulfonamides (n = 2), quinolones (n = 2), fosfomycin (n = 1), macrolides (n = 1), bleomycin (n = 1), and the multi efflux genes (n = 2) (Table 1).

### 3.4. Phenotypic and Genotypic Resistance Correlation in Salmonella Isolates

**Figure 3:**
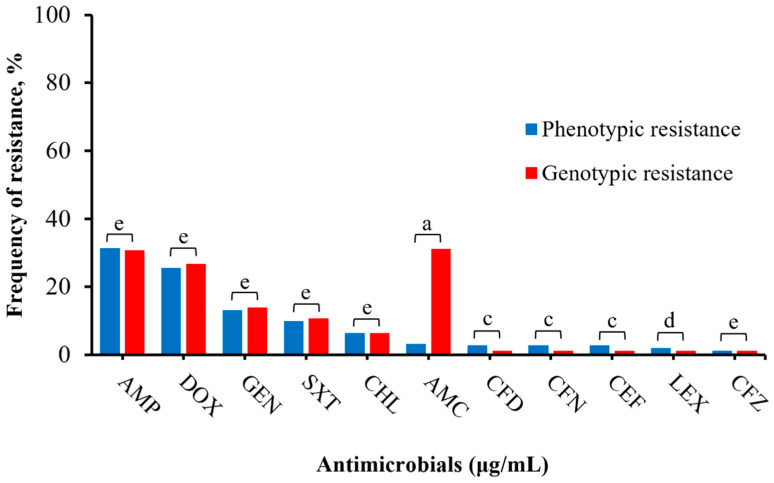
Correlation between phenotype and genotype of *Salmonella* isolates (n = 251). Antimicrobials with no phenotypic resistance profile (imipenem, amikacin, ciprofloxacin, enrofloxacin, marbofloxacin) and nitrofurantoin (no specific resistant genotype) were excluded in this comparison. The total number of phenotypic resistance to genotypic resistance per antimicrobial are AMP: (79/251, 77/251); DOX: (64/251, 67/251), GEN: (33/251, 35/251), SXT: (25/251, 27/251), CHL: (16/251, 16/251), AMC: (8/251, 77/251), CFD: (7/251, 3/251), CFN: (7/251, 3/251), CEF: (7/251, 3/251), LEX: (5/251, 3/251), CFZ: (3/251, 3/251), respectively. The total genotypic resistance was determined by summating the number of isolates positive for at least one common resistant gene per antimicrobial. Results were interpreted as follows: a: 0.01–0.20 slight agreement, b: 0.21–0.40 fair agreement, c: 0.41–0.60 moderate agreement, d: 0.61–0.80 substantial agreement, e: 0.81–1.00 almost perfect or perfect agreement.

Varying levels of phenotypic and genotypic correlations were identified in this study. An isolate was classified as genotypically resistant if it carried at least one resistant gene or mutation related to the specific antibiotic. Notably, there was almost perfect agreement between resistant phenotypes and genotypes for ampicillin (k = 0.98) and for gentamicin, doxycycline, trimethoprim/sulfamethoxazole, and chloramphenicol (k = 0.93–1). Moderate to perfect correlations (k = 0.59–1) were observed for cephalosporins (cephalexin, cefpodoxime, cefovecin, ceftazidime, ceftiofur), whereas only a slight correlation (k = 0.13) was observed for amoxicillin/clavulanic (Figure 3; Table S4).

Interestingly, we observed that isolates with the *bla*_SCO-1_ gene showed intermittent (MIC = 16 μg/mL) to complete resistance (MIC ≥ 32 μg/mL) to amoxicillin/clavulanic acid (Table 1). In this phenotypic and genotypic comparison, we excluded nitrofurantoin (n = 7) due to absence of a corresponding resistance gene and quinolones-resistant genes *gyrA(S83F)* and *parC(T57S)* (n = 249) as no phenotypic resistance was observed.

### 3.5. Plasmid-Mediated AMR Genes

**Figure 4:**
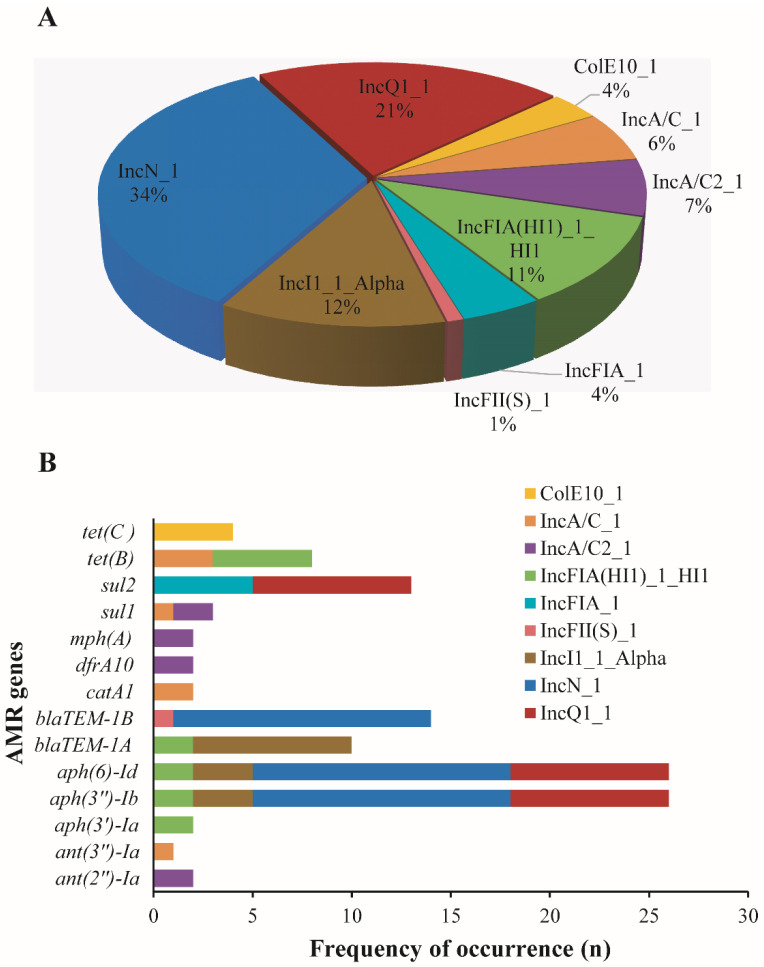
Plasmids harboring antimicrobial resistance genes in *Salmonella* isolates. (**A**): Pie chart highlighting the frequency occurrence of different plasmids (n = 9) associated with antimicrobial resistance (AMR) genes in *Salmonella* isolates investigated in this study. (**B**): Bar chart showing the distribution of different AMR genes (n = 14) on the detected plasmids in different *Salmonella* isolates. Note: These genes were co-located on similar contigs harboring the plasmids.

Plasmid analysis revealed nine different plasmids associated with AMR genes in *Salmonella* isolates. The identified plasmids include ColE10_1, IncA/C_1, IncA/C2_1, IncFIA(HI1)_1_HI1, IncFIA_1, IncFII(S)_1, IncI1_1_Alpha, IncN_1, and IncQ1_1, while IncN_1 (39/115) and (IncQ1_1) (24/115) were the most prevalent plasmids (Figure 4A). Subsequently, we identified fourteen (n = 14) different plasmid-mediated AMR genes across several isolates (Figure 4B).

In these three *S*. Typhimurium isolates, only the IncA/C_1 plasmid of a 417-nucleotide sequence representing a replication protein was found to be associated with AMR genes. These genes were identified as *tetB* for ADRDL-S60, *catA1*, *sul1*, *tet(B)*, and *ant(3″)*-*Ia* for ADRDL-S178 and *catA1* and *tet(B)* for ADRDL-S179. In contrast, two *S*. Muenster isolates did not harbor any plasmid-mediated genes.

### 3.6. Characteristics of bla_SCO-1_ Positive Isolates

**Figure 5:**
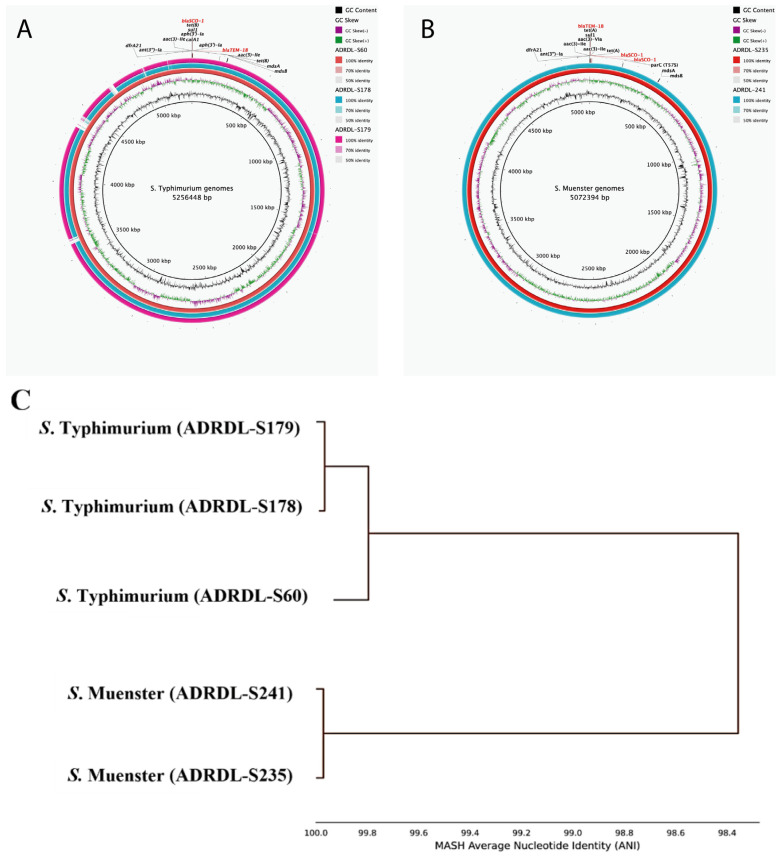
Genomic characterization of *bla*_SCO-1_ -positive *Salmonella* isolates. (**A**): Circular genome representation comparing *S*. Typhimurium genomes (ADRDL-S60, -S178, -S179) that harbored similar AMR genes (n = 11). ADRDL-S60 served as the reference genome (inner ring) and its AMR gene annotation was used for labeling. Each gene was present in two copies except *mdsA* and *mdsB* (**B**): Circular genome representation comparing *S*. Muenster genomes (ADRDL-S235, -S241) with similar AMR genes (n = 11). ADRDL-S235 served as the reference sequence and its AMR gene annotation was used for labeling. Each gene was present in two copies except *mdsA*, *mdsB*, *parC(T57S)*. Legends on the right of each map indicate GC content and GC skew and percentage identity of regions among isolates. The circular maps were generated with BLAST Ring Image Generator (BRIG) software. (https://sourceforge.net/projects/brig/). (**C**): Phylogenetic tree showing the average nucleotide identity of all the *Salmonella* isolates (n = 5), constructed by MASH clustering to determine percentage similarities among isolates. This was calculated using the FastANI pipeline version 1.34 (https://github.com/ParBLiSS/FastANI).

Genomic analysis showed that three *S*. Typhimurium isolates (ADRDL-S60, ADRDL-S178, ADRDL-S179) revealed similar features with 5.2–5.3 Mb genome size, 52% GC content, and 11 antimicrobial resistance (AMR) genes with similar phenotypic characteristics (Figure 5A, Table 1). The *S*. Muenster isolates (ADRDL-S235, ADRDL-S241) exhibited similar phenotypic and genotypic attributes with a 5.1 Mb genome size and 52% GC content (Figure 5B, Table 1). All isolates carried two copies of *bla*_SCO-1_. Phylogenetic analysis revealed three distinct clusters showing 100% nucleotide identity between ADRDL-S178 and ADRDL-S179, and ADRDL-S235 and ADRDL-S241 (Figure 5C).

Amino acid analysis revealed that *bla*_SCO-1_ gene comprised 288 amino acids with a conserved penicillin-binding protein transpeptidase domain. In ADRDL-S178, ADRDL-S179, and ADRDL-S60, one gene copy spanned nucleotides 14,692 to 15,558 and the other from 9831 to 10,697 (except in ADRDL-S178, where it was 1862 to 2728). The ADRDL-S60 isolate had distinct coordinates of 1662 to 2528 and 1703 to 2569.

### 3.7. Comparison of the Genetic Environment of bla_SCO-1_ Gene

**Figure 6:**
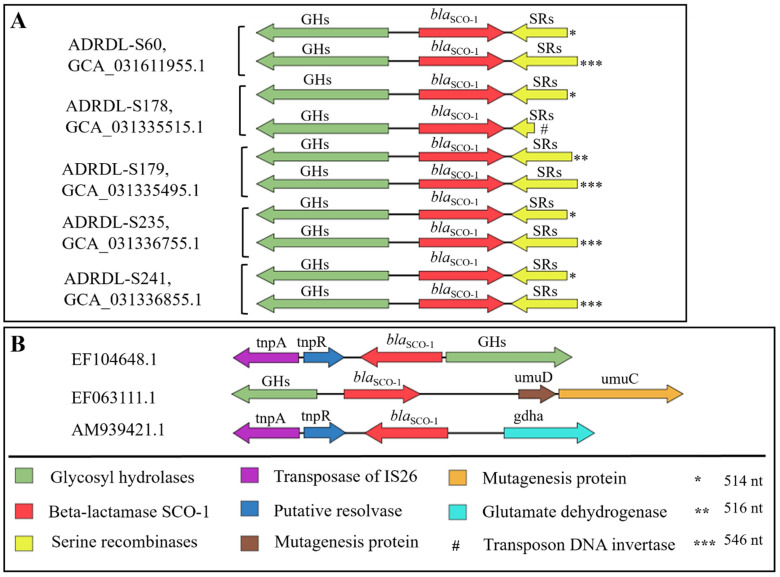
Comparison of the genetic environment of *bla*_SCO-1_ gene. (**A**): Genetic environment of copies of *bla*_SCO-1_ gene (n = 2) found in five *Salmonella* isolates in this study. (**B**): The genetic environment of other isolates was identified to have harbored *bla*_SCO-1_ gene from NCBI database. The direction of the arrows indicates the orientation of the genes, while the length is a representation of their open reading frames (ORFs) All isolates were identified using their accession numbers from NCBI. Asterisks were used to show the varying nucleotide (nt) length of the serine recombinase sequence. Note: Sequence lengths and distances are not drawn to scale.

First of all, the *bla*_SCO-1_ gene in all these five isolates was not associated with any plasmid. The BLASTP analysis with the gene sequence *bla*_SCO-1_ showed a 100% identity with proteins found in isolates with the accession numbers EF104648.1, EF063111.1, and AM939421.1. Furthermore, we comparatively investigated the genetic environment of our isolates and other *bla*_SCO-1_ positive isolates (Figure 6A). Upstream of all copies of the *bla*_SCO-1_ gene in our isolates was occupied by a gene encoding the cellulase family glycosyl hydrolase, comprising 399 amino acids (aa), situated 161 base pairs away from the gene in opposite orientation, while the serine recombinase family protein gene of varying nucleotide lengths (108–546 nucleotides) was located 29 base pairs away from the *bla*_SCO-1_ gene, also in the opposite orientation. Notably, the shortest copy of this gene encoded a specific transposon DNA-invertase of 35 amino acids and was found in the ADRDL-S178 isolate (Figure 6A). Comparison with the identified isolates from NCBI showed some similarities upstream and variations downstream of the *bla*_SCO-1_ gene (Figure 6B).

## 4. Discussion

The occurrence of antimicrobial resistance in *Salmonella* poses a significant threat to public health, despite efforts in recent decades to decrease the use of antibiotics [31,32]. The initiation of the National Antimicrobial Resistance Monitoring Program (NARMS) in 1996 marked the beginning of systematic surveillance for antimicrobial resistance in zoonotic enteric pathogens [33]. However, potential gaps in antimicrobial resistance data since its establishment highlight the need for ongoing and retrospective surveillance in animal populations. A wide range of domestic and wild animals can serve as reservoirs for *Salmonella*, thereby facilitating the dissemination of this pathogen to other animals, environments, and humans [32]. Our retrospective analysis of *Salmonella* isolates recovered from 1982 to 1999 fills a crucial temporal gap, offering insights into resistance trends before NARMS. This historical dataset is a valuable reference for comparing and comprehending recent antimicrobial surveillance data.

The observed phenotypic resistance against the tested antimicrobials showed the highest resistance against ampicillin, which is consistent with previous retrospective reports [34,35]. Ampicillin resistance was initially detected in 1949 but became a significant problem in the early 1980s due to its use in treating salmonellosis, resulting in a notable increase in ampicillin resistance between the 1980s and 2000s [34,35,36]. This shift led to changes in the clinical approach towards the utilization of extended spectrum cephalosporins and quinolones [35]. Our analysis indicated low to no resistance to these antimicrobials. However, recent documentation has shown increasing resistance against both classes of antimicrobials among *Salmonella* serovars recovered from animal and human sources [37,38]. Similarly, high resistance against doxycycline following ampicillin was expected due to its extensive use in animals, owing to its low toxicity and affordability, as reported by Galarce et al. [39].

Serovar-specific variations in resistance frequency were noted in this study, with *S*. Typhimurium displaying the highest resistance among serovars, predominantly distributed in animal hosts. This observation aligns with previous findings that *S*. Typhimurium consistently exhibits greater antimicrobial resistance compared to other common serovars [17,40]. Notably, most of the antimicrobial resistance serovars were recovered from bovine sources, particularly *S*. Typhimurium and *S*. Dublin, which corresponds with previous reports and emphasizes their significance in bovines due to both their zoonotic potential and clinical impact in cattle herds [17,41]. Similarly, in this study, *S*. Typhimurium from equine sources exhibited a high resistance, a trend supported by Spier et al. [42], who documented a high fatality rate of horses associated with *S*. Typhimurium.

In this study, we evaluated the correlation between phenotypic and genotypic resistance, assessing the predictive capability of WGS [43]. Almost perfect to perfect agreement (k = 0.93–1) for ampicillin, doxycycline, chloramphenicol, gentamicin, and trimethoprim/sulfamethoxazole suggests WGS as a reliable predictor. Moderate to substantial agreement (k = 0.59–1) with cephalosporins indicates minor discrepancies. Slight agreement (k = 0.11) for amoxicillin/clavulanic acid may result from clavulanic acid inhibiting beta-lactamase [8]. A false positive was observed in nitrofurantoin, possibly due to unknown resistance mechanisms such as the expression of efflux pumps [44,45]. Conversely, a false negative occurred with quinolone-resistant genes (*gyrA(S83F)*, *parC(T57S)*), indicating phenotypic susceptibility despite the associated genotype, suggesting silenced resistance genes [43].

A significant finding of our study was the identification of *bla*_SCO-1_, an uncommon gene, in *S*. Typhimurium and *S*. Muenster obtained from equine sources between 1989 and 1999, marking the first report of such occurrence in the United States. *bla*_SCO-1_, characterized as a class A beta-lactamase in *Escherichia coli* and *Acinetobacter* species in Greece and Argentina [46,47], was reported in diverse locations and bacterial strains, including in *Salmonella enterica* serovar Livingstone, *Serratia marcescens*, *Aeromonas salmonicida*, *Acinetobacter radioresistens*, and *Klebsiella pneumoniae* in Tunisia, Japan, Switzerland, Antarctica, and Belgium [48,49,50,51,52]. To the best of our knowledge, our recovered isolates harboring this gene as early as 1989 precede those of all other reports.

The *bla*_SCO-1_ positive isolates in this study exhibited resistance to amoxicillin/clavulanic acid, potentially attributed to the *bla*_SCO-1_ gene, as documented by Ruppé et al. [50]. However, this finding contradicts earlier reports suggesting clavulanic acid’s inhibition of narrow-spectrum hydrolysis towards beta-lactams mediated by *bla*_SCO-1_ [46,47]. Nevertheless, we also observed intermediate resistance (MIC = 16 μg/mL) towards amoxicillin/clavulanic acid in two *S*. Muenster isolates harboring this gene. This implies diverse genetic expression or phenotypic impact across strains or species, challenging a uniform interpretation of *bla*_SCO-1_ functional behavior.

The phylogenetic relationship of these five *bla*_SCO-1_ positive isolates in this study revealed distinct clustering patterns, indicating that relationships may be more influenced by genome sequence content, and strain type, than by antimicrobial resistance profiles [53].

While prior studies have reported *bla*_SCO-1_ as plasmid-mediated [46,47,52,54], the non-plasmid association of this gene in this study prompted a closer examination of its genetic environment. *bla*_SCO-1_ occurred as part of a contiguous chromosomal sequence, as indicated by its association with a glycosidase gene upstream, in line with other reports [46,47,50]. The presence of serine recombinases downstream of *bla*_SCO-1_ accompanied by transposon DNA invertase could facilitate site-specific DNA rearrangement, potentially enhancing the mobility of this gene [47,50,55,56].

Prior studies have also emphasized the lack of homology across these plasmids due to the absence of the *bla*_SCO-1_ gene in highly similar plasmids [46,47,52,54]. This raises questions about the intrinsic or acquired nature of this resistance gene.

It is important to highlight that *bla*_SCO-1_ has predominantly been identified in clinical isolates obtained from both human and animal hosts, including isolates associated with nosocomial outbreaks [46,48,49,50]. In our study, the serovars *S*. Muenster and *S*. Typhimurium, which harbor this gene, have previously been implicated in human salmonellosis outbreaks originating from zoonotic sources [57,58].

While the in silico PlasmidFinder web tool utilized in this study has been validated to identify known plasmids by searching for conserved replicon sites and comparing them to a curated database of plasmid replicons, it was designed to identify at least 80% nucleotide identity with those currently included in the database and will not adequately cover plasmid diversity outside this scope [25]. Nevertheless, this method was sufficient for other authors who reported *bla*_SCO-1_ as plasmid-mediated [52].

Overall, our study contributes to the ongoing discourse on the genomic landscape of antibiotic resistance in *Salmonella*, providing crucial insights for public health efforts. The complexities surrounding *bla*_SCO-1_ identified in this study warrant further investigation to decipher its implications for the evolution of antibiotic resistance.

## Figures and Tables

**Table 1:** Antimicrobial resistance genes (AMGs) identified in *Salmonella* isolates.

Antimicrobial Class	AMG	Number of Isolates Harboring the AMG
Aminoglycosides	*aph(6)-Ic*	16
	*aph(6)-Id*	49
	*aac(3)-Via*	4
	*aph(3′)-IIa*	16
	*aadA2*	17
	*aadA15*	1
	*aac(3)-IId*	11
	*aac(3)-IIe*	5
	*ant(2″)-Ia*	18
	*aph(3′)-Ia*	55
	*aadA7*	1
	*aph(3″)-Ib*	52
	*ant(3″)-Ia*	44
Beta-lactams	*bla*_TEM-1A_	8
	*bla*_TEM-1B_	71
	*bla*_CARB-2_	1
	*bla*_CMY-2_	3
	*bla*_OXA-2_	1
	*bla*_SCO-1_	5
Tetracycline	*tet(A)*	39
	*tet(B)*	24
	*tet(C)*	3
	*tet(G)*	1
Phenicol	*catA1*	12
	*cmlA1*	1
	*floR*	4
Sulfonamide	*sul1*	66
	*sul2*	22
Trimethoprim	*dfrA12*	9
	*dfrA21*	7
	*dfrA10*	11
Quinolone	*gyrA (S83F)*	2
	*parC (T57S)*	128
Fosfomycin	*fosA7*	23
Macrolide	*mph(A)*	12
Bleomycin	*ble*	16
Multi-drug efflux	*mdsA*	249
	*mdsB*	249

## Data Availability

The data presented in this study are openly available online in NCBI data repository at: https://www.ncbi.nlm.nih.gov/bioproject/ (accessed on 25 February 2024) with a BioProject accession number: PRJNA280335. The isolates can be found with identifiers ranging from ADRDL S1—ADRDL S251.

## Supplementary Materials

The following supporting information can be downloaded at: https://www.mdpi.com/article/10.3390/microorganisms12030528/s1, Table S1: Frequency of distribution of *Salmonella* serovars (n = 251) across 11 animal host species; Table S2: Antimicrobial susceptibility profile of *Salmonella* isolates (n = 251) in this study; Table S3: Antimicrobial resistance in 101 *Salmonella* isolates across 11 serovars and 11 animal species exhibiting resistance to at least one antimicrobial tested; Table S4: Correlation between phenotype and genotype of *Salmonella* isolates (n = 251) in this study.
