# Supplementary material for: A Retrospective Analysis of Salmonella Isolates across 11 Animal Species (1982–1999) Led to the First Identification of Chromosomally Encoded blaSCO-1 in the USA
Source: Microorganisms. 2024 Mar 6;12(3):528. doi: 10.3390/microorganisms12030528 (PMC10974302; doi:10.3390/microorganisms12030528)
Supplement: Supplementary file 1 [file microorganisms-12-00528-s001.zip › microorganisms-2888944-supplementary.pdf]

Table S1: Frequency of distribution of *Salmonella* serovars (n = 251) across 11 animal host species

Serovars (number of the isolates)	Number of isolates per animal host species										
	Avian	Bovine	Canine	Deer	Equine	Feline	llama	Ovine	Porcine	Reptile	Rhea
Agona (18)	0	7	0	0	11	0	0	0	0	0	0
Amsterdam (1)	0	0	0	0	1	0	0	0	0	0	0
Anatum (23)	0	13	0	0	10	0	0	0	0	0	0
Berta (3)	0	0	0	2	1	0	0	0	0	0	0
Braenderup (2)	0	0	0	0	2	0	0	0	0	0	0
Cerro (1)	0	0	0	0	1	0	0	0	0	0	0
Choleraesuis (3)	0	0	0	0	0	0	0	0	3	0	0
Dublin (21)	0	21	0	0	0	0	0	0	0	0	0
Enteritidis (6)	0	1	1	0	2	0	0	2	0	0	0
Florida (1)	0	0	0	0	1	0	0	0	0	0	0
Give (8)	0	7	0	0	1	0	0	0	0	0	0
Havana (2)	0	0	0	0	1	1	0	0	0	0	0
I 4,[5],12:i:- (3)	0	1	0	0	2	0	0	0	0	0	0
I 47:z4,z23:- (1)	0	1	0	0	0	0	0	0	0	0	0
IIIa -:z4,z23:- (1)	0	0	0	0	1	0	0	0	0	0	0
IIIb 48:l,v:1,5,(7) (1)	0	0	0	0	0	0	0	0	0	1	0
Infantis (2)	0	1	1	0	0	0	0	0	0	0	0
Javiana (2)	0	0	0	0	2	0	0	0	0	0	0
Lexington (1)	0	1	0	0	0	0	0	0	0	0	0
Lille (1)	0	0	0	0	1	0	0	0	0	0	0
Mbandaka (8)	0	5	0	0	3	0	0	0	0	0	0
Meleagridis (5)	0	5	0	0	0	0	0	0	0	0	0
Montevideo (4)	0	0	0	0	4	0	0	0	0	0	0
Muenchen (7)	0	0	1	0	6	0	0	0	0	0	0
Muenster (8)	0	0	1	0	7	0	0	0	0	0	0

Newport (11)	1	2	0	0	6	1	1	0	0	0	0
Nottingham (1)	0	0	0	0	1	0	0	0	0	0	0
Nyanza (1)	0	0	0	0	0	0	0	0	0	1	0
Oranienburg (1)	0	0	0	0	1	0	0	0	0	0	0
Saintpaul (2)	0	0	0	0	2	0	0	0	0	0	0
Schwarzengrund (1)	1	0	0	0	0	0	0	0	0	0	0
Senftenberg (2)	0	1	0	0	0	1	0	0	0	0	0
Tennessee (3)	0	0	0	0	1	0	0	0	0	0	2
Typhimurium (91)	2	34	6	1	45	0	0	0	2	0	1
Uganda (2)	0	2	0	0	0	0	0	0	0	0	0
Worthington (3)	0	0	0	0	3	0	0	0	0	0	0
**Total (251)**	**4**	**102**	**10**	**3**	**116**	**3**	**1**	**2**	**5**	**2**	**3**

Table S2. Antimicrobial susceptibility profile of *Salmonella* isolates (n=251) in this study

Antibiotics	Abbreviation	Phenotype		
		Resistance (%)	Intermediate (%)	Susceptible (%)
Ampicillin	AMP	79 (31.5)	0 (0.0)	172 (68.5)
Doxycycline	DOX	64 (25.5)	1 (0.4)	186 (74.1)
Gentamicin	GEN	33 (13.1)	0 (0.0)	218 (86.9)
Trimethoprim/sulfamethoxazole	SXT	25 (10.0)	0 (0.0)	226 (90.0)
Chloramphenicol	CHL	16 (6.4)	4 (1.6)	231 (92.0)
Amoxicillin/clavulanic acid	AMC	8 (3.2)	8 (3.2)	235 (93.6)
Cefpodoxime	CPD	7 (2.8)	0 (0.0)	244 (97.2)
Nitrofurantoin	NIT	7 (2.8)	32 (12.7)	212 (84.5)
Cefovecin	CFN	7 (2.8)	2 (0.8)	242 (96.4)
Ceftiofur	CEF	7 (2.8)	1 (0.4)	243 (96.8)
Cephalexin	LEX	5 (2.0)	0 (0.0)	246 (98.0)
Ceftazidime	CFZ	3 (1.2)	0 (0.0)	248 (98.8)
Ciprofloxacin	CIP	0 (0.0)	3 (1.2)	248 (98.8)
Enrofloxacin	EF	0 (0.0)	1 (0.4)	250 (99.6)
Imipenem	IPM	0 (0.0)	0 (0.0)	251 (100.0)
Amikacin	AMK	0 (0.0)	0 (0.0)	251 (100.0)
Marbofloxacin	MAR	0 (0.0)	0 (0.0)	251 (100.0)

Table S3. Antimicrobial resistance in 101 *Salmonella* isolates across 11 serovars and 11 animal species exhibiting resistance to at least one antimicrobial tested

***Salmonella* Serovars**	**Animal host species**											**Total (%)**
	**Avian**	**Bovine**	**Canine**	**Deer**	**Equine**	**Feline**	**Ilama**	**Ovine**	**Porcine**	**Reptile**	**Rhea**	
Typhimurium	1 (1.0)	28 (27.7)	5 (5.0)	1 (1.0)	15 (14.9)	0 (0.0)	0 (0.0)	0 (0.0)	0 (0.0)	0 (0.0)	0 (0.0)	**50 (49.5)**
Dublin	0 (0.0)	16 (15.8)	0 (0.0)	0 (0.0)	0 (0.0)	0 (0.0)	0 (0.0)	0 (0.0)	0 (0.0)	0 (0.0)	0 (0.0)	**16 (15.8)**
Agona	0 (0.0)	1 (1.0)	0 (0.0)	0 (0.0)	8 (7.9)	0 (0.0)	0 (0.0)	0 (0.0)	0 (0.0)	0 (0.0)	0 (0.0)	**9 (8.9)**
Muenster	0 (0.0)	0 (0.0)	0 (0.0)	0 (0.0)	6 (5.9)	0 (0.0)	0 (0.0)	0 (0.0)	0 (0.0)	0 (0.0)	0 (0.0)	**6 (5.9)**
Anatum	0 (0.0)	1 (1.0)	0 (0.0)	0 (0.0)	4 (4.0)	0 (0.0)	0 (0.0)	0 (0.0)	0 (0.0)	0 (0.0)	0 (0.0)	**5 (5.0)**
Enteritidis	0 (0.0)	1 (1.0)	0 (0.0)	0 (0.0)	2 (2.0)	0 (0.0)	0 (0.0)	2 (2.0)	0 (0.0)	0 (0.0)	0 (0.0)	**5 (5.0)**
Newport	0 (0.0)	2 (2.0)	0 (0.0)	0 (0.0	2 (2.0)	1 (1.0)	0 (0.0)	0 (0.0)	0 (0.0)	0 (0.0)	0 (0.0)	**5 (5.0)**
Mbandaka	0 (0.0)	2 (2.0)	0 (0.0)	0 (0.0)	0 (0.0)	0 (0.0)	0 (0.0)	0 (0.0)	0 (0.0)	0 (0.0)	0 (0.0)	**2 (2.0)**
Choleraesuis	0 (0.0)	0 (0.0)	0 (0.0)	0 (0.0)	0 (0.0)	0 (0.0)	0 (0.0)	0 (0.0)	1 (1.0)	0 (0.0)	0 (0.0)	**1 (1.0)**
Meleagridis	0 (0.0)	1 (1.0)	0 (0.0)	0 (0.0)	0 (0.0)	0 (0.0)	0 (0.0)	0 (0.0)	0 (0.0)	0 (0.0)	0 (0.0)	**1 (1.0)**
Worthington	0 (0.0)	0 (0.0)	0 (0.0)	0 (0.0)	1 (1.0)	0 (0.0)	0 (0.0)	0 (0.0)	0 (0.0)	0 (0.0)	0 (0.0)	**1 (1.0)**
**Total** (%)	**1 (1.0)**	**52 (51.5)**	**5 (5.0)**	**1 (1.0)**	**38 (37.6)**	**1 (1.0)**	**0 (0.0)**	**2 (2.0)**	**1 (1.0)**	**0 (0.0)**	**0 (0.0)**	**101 (100)**

Table S4. Correlation between phenotype and genotype of *Salmonella* isolates (n=251)

**Antimicrobials**	**Phenotypically resistance**		**Phenotypically susceptible**		**Kappa value (k)**	**PR (%)**	**GR (%)**
	**Genotypically resistance**	**Genotypically susceptible**	**Genotypically resistance**	**Genotypically susceptible**			
Ampicillin (AMP)	77	2	0	172	0.98	79 (31.5)	77 (30.7)
Amoxicillin/clavulanic acid (AMC)	7	1	70	173	0.11	8 (3.2)	77 (30.7)
Cephalexin (LEX)	3	2	0	246	0.74	5 (2.0)	3 (1.2)
Cefpodoxime (CFD)	3	4	0	244	0.59	7 (2.8)	3 (1.2)
Cefovecin (CFN)	3	4	0	244	0.59	7 (2.8)	3 (1.2)
Ceftazidime (CFZ)	3	0	0	248	1.00	3(1.2)	3 (1.2)
Ceftiofur (CEF)	3	4	0	244	0.59	7 (2.8)	3 (1.2)
Gentamicin (GEN)	32	1	3	215	0.93	33 (13.1)	35 (13.9)
Doxycycline (DOX)	64	0	3	184	0.96	64 (25.5)	67 (26.7)
Chloramphenicol (CHL)	16	0	0	235	1.00	16 (6.4)	16 (6.4)
Trimethoprim/sulfamethoxazole (SXT)	25	0	2	224	0.95	25 (10.0)	27 (10.8)

PR and GR denote total phenotypic resistance and total genotypic resistance respectively
